# Association between Maternal and Child Nutritional Status in Hula, Rural Southern Ethiopia: A Cross Sectional Study

**DOI:** 10.1371/journal.pone.0142301

**Published:** 2015-11-20

**Authors:** Canaan Negash, Susan J. Whiting, Carol J. Henry, Tefera Belachew, Tewodros G. Hailemariam

**Affiliations:** 1 School of Public Health, Wolaita Sodo University, Wolaita, Ethiopia; 2 College of Pharmacy and Nutrition, University of Saskatchewan, Saskatoon, Canada; 3 Department of Population & Family Health, Jimma University, Jimma, Ethiopia; University of Bremen, GERMANY

## Abstract

**Background:**

Maternal and child under nutrition is highly prevalent in low-income and middle-income countries, resulting in substantial increases in mortality and overall disease burden. The aim of this baseline survey was to determine the association between selected maternal characteristics, maternal nutritional status and children’s nutritional status.

**Methods and Findings:**

A survey with a cross sectional design was conducted between September and October 2012 in Hula, Ethiopia. The study subjects were 197 mothers of children between the ages of 6 and 23 months. Weight and height (mothers) or recumbent length (children) were measured using calibrated, standardized techniques. Seven percent of children were below -2 weight for height Z score (WHZ), 11.5% were below -2 height for age Z score (HAZ) and 9.9% were below -2 weight for age Z score (WAZ). Maternal anthropometrics were associated with child nutritional status in the bivariate analysis. Maternal BMI (*r* = 0.16 *P* = 0.02) and educational status (*r* = 0.25 *P* = 0.001) were correlated with WHZ of children while maternal height (*r* = 0.2 *P* = 0.007) was correlated with HAZ of children. After multivariate analysis, children whose mothers had salary from employment had a better WHZ score (*P* = 0.001) and WAZ score (*P*<0.001). Both maternal BMI and maternal height were associated with WHZ (*P* = 0.04) and HAZ (*P* = 0.01) score of children.

**Conclusion:**

Having a mother with better nutritional status and salaried employment is a benefit for the nutritional status of the child. The interrelationship between maternal and child nutritional status stresses the value of improving maternal nutritional status as this should improve both maternal and child health outcomes. Therefore strategies to improve nutritional status of children should also include improving the nutritional status of the mother and empowering her financially.

## Introduction

Maternal and child under nutrition is the underlying cause of 3·5 million deaths, 35% of the disease burden in children younger than 5 years and 11% of total global disability-adjusted life-years (DALYs). It is highly prevalent in low-income and middle-income countries, resulting in substantial increases in mortality and overall disease burden [1]. New evidence reinforces the importance of the maternal nutritional status, both for the health of the mother and for ensuring healthy fetal growth and development. It also strengthens the case for a continued focus on the crucial 1000 day window of life [2].

Women’s employment increases household income, with consequent benefit to household nutrition in general and the woman’s nutritional status in particular. Employment may increase women’s status and power, and may bolster a woman’s preference to spend her earnings on health and nutrition [3]. Its effect on child welfare is of enormous importance to development policy as healthy child development and improved economic opportunity for women are two general goals of development agencies worldwide [4]. A study from Malaysia showed that children of unemployed mothers were severely wasted compared to those who were employed [5].

Research indicates that there is a strong linkage between maternal education and children’s health. Children born to less educated women suffer more from malnutrition which manifests as underweight, wasting and stunting in children. Maternal education has been associated with nutrition outcomes among children in studies in various settings including Bolivia [6] and Kenya [7]. Therefore the aim of this study was to assess the relationship between selected maternal characteristics, maternal nutritional status and children’s nutritional status, in Ethiopia.

## Materials and Methods

### Study area and design

This study uses the baseline survey of an intervention study conducted from September 2012 to March 2013, in Hula Woreda, Southern Nations Nationalities and Peoples’ Region (SNNPR), Ethiopia [8]. This baseline survey with a cross sectional design was conducted between September and October 2012. Hula is mostly rural (94%), with 60% cultivable land. The study subjects were 197 mothers of children between the ages of 6 and 23 months living in two kebeles (Titicha and Debicha) of Hula Woreda. Only caregivers who had been residents of the study area for more than 6 months and who gave consent were eligible for the study. To select mother–child pairs, small areas (gots) were randomly chosen in each kebele.

### Measurement

A baseline survey was performed with the use of a pretested, structured questionnaire to assess socio demographic characteristics of the family and maternal characteristics. The questionnaire was developed in English using process validation and then translated into the Sidamigna language. Wealth was estimated using a principal component analysis and which had 19 items with Cronbach’s alpha of 0.7. Measurements of weight and height from both mother and children were taken by trained staff during the baseline survey.

Anthropometric measurements were performed using calibrated equipment and standardized techniques. Weight was measured to the nearest 0.1 kg with an electronic scale (Seca 770) with the children wearing a light shirt and without shoes. Recumbent length was measured to the nearest 0.1 cm with a length-measuring board. All anthropometric data were collected by the principal investigator.

### Data analysis

The data were entered, checked for missing values and outliers, and analyzed with SPSS, version 16.1. The weights and heights of the children were converted to z-scores with WHO Anthro Software. Significant Pearson’s correlation coefficients guided selection of education, demographic, socio-economic and anthropometric variables for linear regression models. To identify the predictors of child nutritional status only variables that were significantly associated at (p < .2) in the bivariate analyses were entered into multivariable linear regression models. In all tests, P < .05 was considered to indicate statistical significance.

### Ethical clearance

Before data collection, permission was obtained from the Hawassa University Ethical Review Committee. The local authorities were informed about the purpose of the study and its objectives. Prior to entry into the study, the mothers gave oral consent after the benefits of the study had been explained to them. Sick and severely malnourished children were referred to health facilities and advice was given to their parents.

## Result

### Socio economic and socio demographic factors

Overall, the response rate of study participants was 98%. Almost all (99%) mothers in the study area were married and 98% were Sidama by ethnicity. As shown in Table 1 most mothers (81%) were followers of a Protestant religion and 61.9% had no formal education. The majority (81%) of families in the study area were reliant on farming for income and 32% of the mothers were farmers. They were living in houses made of a bamboo wall (75.1%), thatched grass roof (75.6%) and drinking water from a community tap water source (60.4%). Although 65.5% households were in the middle level for wealth status, only 14.2% indicated that children had priority for food at mealtime.

**Table 1:** Socio demographic and household characteristics.

Variables (n = 197)		Frequency	Percent (%)
Religion	Protestant	160	81.2
	Orthodox	4	2.0
	Muslim	8	4.1
	Catholic	25	12.7
Educational Status	No formal education	122	61.9
	Primary	58	29.4
	Secondary	11	5.6
	Preparatory	4	2.0
	Others	2	1.0
Occupation	Unemployed	128	64.9
	Farmer	62	31.5
	Employed	7	3.6
Head of the household	Mother	10	5.1
	Father	187	94.9
Water source	Tap water	119	60.4
	Non tap water	78	39.6
Wall of the House	Wood and mud	49	24.9
	Bamboo	148	75.1
Roof of the House	Corrugated iron sheet	25	12.7
	Thatched grass	149	75.6
	Bamboo	23	11.7
Main income source	Trade	21	10.7
	Farming	160	81.2
	Salary from employment	16	8.1
Meal priority	Men	149	75.6
	Women	5	2.5
	Children	28	14.2
	Others	15	7.6
Wealth	Low level	63	31.9
	Medium level	129	65.5
	High level	5	2.5
	No	6	3.1
Mother listens to radio	Yes	35	17.8
	No	162	82.2
Mother watches TV	Yes	7	3.6
	No	190	96.5

Ninety five percent of the households were male headed. Close to 18% of mothers listened to radio while 4% watched Television.

### Mother-child nutritional status

As Table 2 shows, most children (65%) were 12–24 months old. Seven percent of the children were below -2 weight for height Z score (WHZ), 11.5% were below -2 height for age Z score (HAZ) and 9.9% were below -2 weight for age Z score (WAZ). Almost half of the mothers (48%) were 25–29 years old, and 14.2% were below a body mass index of 18.5 kg/m^2^ (BMI).

**Table 2:** Mothers' and children's anthropometry.

Variables (n = 197)		Frequency	Percent
Children’s sex	Male	95	48.2
	Female	102	51.8
Children’s age	6–8	35	17.8
	9–11	34	17.3
	12–24	128	65
Children’s Nutritional status	Wasting (<-2WHZ)	13	6.8
	Stunting (<-2 HAZ)	22	11.5
	Underweight (<-2WAZ)	19	9.9
Mothers’ age	15–19 years	5	2.5
	20–24 years	38	19.3
	25–29 years	95	48.2
	30–34 years	45	22.8
	≥35 years	14	7.1
Mothers’ height	<145 cm	32	16.2
	145–149.9 cm	28	14.2
	150–154.9 cm	48	24.4
	155–159.9 cm	61	31
	> = 160 cm	28	14.2
Mothers’ BMI	<18.5 kg/m^2^	28	14.2
	18.5–24.9 kg/m^2^	166	84.3
	25–29.9 kg/m^2^	1	0.5
	30–34.9 kg/m^2^	2	1

WHZ: weight for height Z score; HAZ: height-for-age z-score; WAZ: weight-for-age z-score Mothers’ BMI: Mothers’ body mass index.

Table 3 shows correlations between maternal characteristics and child nutritional status. Both maternal BMI (*r* = 0.16 *P* = 0.02) and educational status (*r* = 0.25 *P* = 0.001) were correlated with WHZ of children Table 3. Maternal height (*r* = 0.2 *P* = 0.007) was correlated with HAZ of children.

**Table 3:** Pearson correlation coefficient for mother-child variables (n = 197).

	Family size	Edu-cational Status	Main income source	Sex	BMI	Maternal height	Water source	Maternal weight	WHZ	HAZ
Family size	1									
Educational Status	.413**									
Main income source	0.071	.144*								
Sex	0.107	0.133	0.061							
BMI	-0.025	0.136	-0.045	-0.051						
Maternal height	-0.064	0.008	0.1	0.099	-0.074					
Water source	-0.01	0.019	0	0.013	-0.022	0.063				
Maternal weight	0.073	.216**	0.082	0.029	.558**	.470**	0			
WHZ	.162*	.236**	0.126	.147*	.165*	-0.016	-0.055	0.125		
HAZ	0.05	-0.002	0.135	.158*	-0.006	.193**	.147*	0.101	-0.099	
WAZ	.144*	.169*	.189**	.223**	0.116	0.129	0.072	.156*	.656**	.679**

**. Correlation is significant at the 0.01 level (2-tailed).

*. Correlation is significant at the 0.05 level (2-tailed). WHZ: weight for height Z score; HAZ: height-for-age z-score; WAZ: weight-for-age z-score Mothers’ BMI: Mothers’ body mass index

### Predictors of child nutritional status

A stepwise regression analysis was used to identify the predictors of WHZ, HAZ and WAZ Table 4. All socio demographic and maternal anthropometric variables that were significant at P≤0.2 were entered in to the models. Salary from an employment (either from government work or wage) and normal BMI (18.5 to 24.9kg/m^2^) significantly predicted WHZ score of children. Being an illiterate mother was negatively (P = 0.03) associated with WHZ score of children. A significant association was found between maternal height and HAZ. Even though, tap water source was associated with HAZ in the bivariate analysis, it did not become significant in the multivariate analysis. Moreover, salary from employment and WAZ were associated with each other.

**Table 4:** Predictors of children’s WHZ, HAZ and WAZ.

	β coefficient	95% CI	P- value
**WHZ (R** ^**2**^ **= 14.1%)**			
(Constant)	-0.621	-1.076; -0.166	0.008
Salary from employment	1.048	0.456; 1.641	0.001
Mothers’ BMI (18.5to24.9kg/m^2^)	0.627	0.198; 1.056	0.004
Educational status (illiterate)	-0.365	-0.698; -0.032	0.032
**HAZ (R** ^**2**^ **= 10.1%)**			
(Constant)	-0.386	-0.776; 0.004	0.052
Maternal height (3^rd^ tertile: ≥157.1 cm)	0.630	0.95; 1.165	0.021
Salary from employment	1.158	0.174; 2.143	0.021
Maternal Age (≥35 years)	-1.079	-2.037;-0.120	0.028
**WAZ (R** ^**2**^ **= 16.1%)**			
(Constant)	-0.299	-0.524; -0.075	0.009
Salary from employment	1.333	0.765; 1.9	<0.001
Sex (male)	-0.369	0.684; 0.055	0.022
Maternal Age (≥35 years)	-0.653	-1.26; -0.047	0.035

WHZ: weight for height Z score; HAZ: height-for-age z-score; WAZ: weight-for-age z-score Mothers’ BMI: Mothers’ body mass index, (Salary from employment (Yes = 1, No = 0), Sex (Male = 1, Female = 0)).

## Discussion

This baseline survey was part of an intervention study undertaken in the rural southern part of Ethiopia [8]. The results of this study show maternal anthropometry predicted children’s nutritional status, i.e., normal maternal BMI (18.5 to 24.9kg/m^2^) predicted weight for height Z (WHZ) score. This effect could be explained as being due to mothers in the high wealth classification, who had higher BMI than those who were in middle and low wealth classification (P<0.001). A study done in Nigeria showed that maternal BMI correlated with income and educational status [9]. Further, according to the 2011 Ethiopia demographic and health survey (EDHS), wasting (weight for height <-2SD) was most common in the children of thin mothers (whose BMI is less than 18.5), in children whose mothers have no education, and in children in the lowest wealth quintiles [10]. In a study done in a daycare center in Brazil, both maternal weight and height had a significant impact on children’s nutritional status [11].

Similarly in this study, maternal educational status was also a positive predictor for WHZ score of children. This may be explained as the more mothers obtain education, the more they will visit a health center and get nutritional advice from health professionals. Another explanation may be that as they are educated, they will have jobs that pay well and this will increase mothers’ purchasing power, which is consistent with a study done in Bolivia [6].

Having a salary from employment (either from wage or government work) was a strong predictor for all of the following: weight for height Z (WHZ) score, height for age Z (HAZ) score and weight for age Z (WAZ) score of children. A cross-sectional study from Brazil [12] showed lower mean HAZ among children whose mothers presented a lower household income (p<0.05). However, after adjustment for maternal age, educational level, household income and other factors, children whose mothers’ height was <145 cm had a 1.2 lower HAZ than those whose mothers were ≥160 cm tall (P for trend <0.0001) [12]. In addition to the wealth of a household, it may also be important for mothers to have their own money so that they can buy a variety of food, thereby improving diet diversity. Thus empowering women financially could be an important step, to take in improving child nutritional status. Results consistent with this hypothesis were observed in a study in a low income neighborhood in Nicaragua where mothers’ wealth correlated with children’s WAZ and WHZ, but maternal employment particularly affected WHZ score of their children [4]. Moreover, a research conducted on Malaysian mothers, shows positive correlation between working hours and weight of their young children [5]. In a study conducted in Nigeria, maternal work status was associated with significant and positive impact on child growth in regions with high levels of malnutrition [13].

Maternal height (3^rd^ tertile: ≥157.1 cm) predicted height for age Z (HAZ) score of children in our study. This is probably due to maternal height influencing offspring linear growth over the growth period [14]. A study conducted in Brazilian children revealed that mothers in the highest tertile of height had children whose HAZ was significantly higher compared with those of children whose mothers were in the lowest height tertile, after adjusting for socioeconomic status [15]. Our finding agrees with the results observed in rural Kenya, in which HAZ score of children significantly increased with an increase in maternal height, wherein an increase in maternal height [OR = 0.95 (CI: 0.91–0.99)] was significantly associated with lower odds of stunting [16]. In our study, being male was negatively associated with height for age Z score. In a meta-analysis of 16 demographic and health survey of sub-Saharan countries, the mean Z-scores for males were consistently lower than for females with the differences statistically significant in 12 out of 16 studies [17].

In the present study, older maternal age (≥35 years) was negatively associated with HAZ and WAZ score of children. It could be that older mothers had less health seeking behaviors compared to younger mothers. However, it may not be a linear relationship, as an in-depth analysis of the Ethiopia demographic and health survey 2005 provides evidence that the youngest (15–19) and oldest (35–49) mothers are both less likely to have health seeking behaviors than mothers in the reference category (20–34) [18]. According to the World Bank, malnutrition is lowest among children born to mothers in their mid-late twenties. Mothers in their teens and their 30s, especially the late 30s, are significantly more likely to have children suffering from malnutrition [19].

### Limitation

The cross sectional nature of this study makes causal relationships between maternal and child nutritional status less probable. Moreover, since the study was from one area of Ethiopia, it could be difficult to make generalizations. However, since it was conducted in rural part of the country, it will give us an initial point for interventions and further studies in similar context.

## Conclusion

In conclusion, children born from mothers who were educated and have salary from employment had a better nutritional status. Normal maternal BMI was associated with WHZ score of children, while maternal height was associated with children’s HAZ score. The interrelationship between maternal and child nutritional status stresses the value of improving maternal nutritional status to make better both maternal and child health outcomes.
